# Smoking cessation counselling patterns in cancer patients – survey of Lebanese physicians

**DOI:** 10.3332/ecancer.2024.1699

**Published:** 2024-04-25

**Authors:** Jad Najdi, Mariana El Hawa, Adnan El-Achkar, Nour Naji, Talar Telvizian, Maya Romani, Albert El Hajj, Deborah Mukherji

**Affiliations:** 1 Division of Urology, Department of Surgery, American University of Beirut Medical Center, Beirut, Lebanon; 2 Department of Dermatology, American University of Beirut Medical Center, Beirut, Lebanon; 3 Department of Hematology and Oncology, Johns Hopkins Medical Center, Baltimore, Maryland, USA; 4 Department of Internal Medicine, Lankenau Medical Center, Wynnewood, PA, USA; 5 Department of Family Medicine, American University of Beirut Medical Center, Beirut, Lebanon; 6 Division of Hematology and Oncology, Department of Internal Medicine, American University of Beirut Medical Center, Beirut, Lebanon; a https://orcid.org/0000-0001-6224-894X; b https://orcid.org/0000-0002-3656-4217; c https://orcid.org/0009-0004-4761-7435; d https://orcid.org/0000-0002-6407-6501; e https://orcid.org/0000-0001-5437-0128; f https://orcid.org/0000-0002-0043-7195; g https://orcid.org/0000-0002-3635-0083

**Keywords:** counselling, neoplasms, physicians, smoking cessation, tobacco

## Abstract

**Introduction:**

Tobacco smoking is a known risk factor for cancer development and smoking cessation can lower this risk and improve outcomes in some cancer patients. Despite that, many cancer patients do not quit smoking after a cancer diagnosis, and smoking cessation counselling is still not routinely provided in cancer care. The aim of this study is to examine patterns in smoking cessation counselling to cancer patients by their treating physicians.

**Methods:**

A self-administered, web-based (mobile-friendly), anonymous questionnaire was developed on LimeSurvey and sent by e-mail to Lebanese physicians of different specialties between June 2020 and January 2022. Data were analysed using SPSS and associations between the different items were determined using the χ^2^ test.

**Results:**

A total of 146 physicians filled out the questionnaire. Almost all physicians ask cancer patients about their smoking status, but only 45.9% provide smoking cessation counselling, and only 24% refer patients to smoking cessation counselling programs. Only 27.4% of all respondents have received formal smoking cessation training, and only 27.4% feel capable of providing smoking cessation counselling in their clinic. Specifically, family medicine physicians were more likely to provide smoking cessation counselling in the clinic (69%), more likely to refer patients to a smoking cessation counselling program (44%), and more likely to have received formal smoking cessation counselling training (67%) and more likely to feel capable of providing smoking cessation counselling (93%). Lack of training, lack of knowledge of available programs and the lack of availability of enough programs are leading obstacles contributing to low rates of smoking cessation counselling in cancer patients as reported by the physicians.

**Conclusion:**

Our data reveals a deficiency in smoking cessation counselling and referral of cancer patients to smoking cessation counselling programs in our region. This highlights the need for dedicated smoking cessation counselling training for practicing physicians and physicians in training.

## Background/Introduction

Tobacco smoking is an alarming risk factor implicated in the development of multiple types of cancer including lung [1], head and neck [2] and bladder cancer [3] among others. Interestingly, smoking was reported to be responsible for almost 50% of all bladder cancer cases and 40% of all bladder cancer deaths in various populations [4].

Several studies have assessed the negative impact of tobacco smoking on both the quality of life and continuity of care in cancer patients. Smoking was found to have a negative effect on pulmonary and immune functions if it is continued after cancer diagnosis [5, 6]. Moreover, a decrease in response to adjuvant chemotherapy was noted in some patients [7]. This was explained by the fact that tobacco smoke induces cytochrome P450 enzymes which metabolise some chemotherapeutic drugs thus lowering their plasma concentrations [7]. Smoking was also shown to worsen radiation and chemotherapy side effects [8, 9]. Furthermore, the risk of a second malignancy at the same site or a different one increases if smokers diagnosed with cancer (smoking-related or non-smoking-related) continue to smoke [10]. Moreover, smoking was found to contribute to a poorer quality of life in cancer patients who continue to smoke [6].

Despite the above-listed risks imposed by smoking, estimates indicate that between 35% and 62% of cancer patients do not quit smoking in the year following diagnosis [11]. For instance, in one study, it was shown that up to 83% of smokers diagnosed with lung cancer continued to smoke after diagnosis [12]. The diminished rate of smoking cessation among cancer patients has not been a priority for many physicians. Often, a cancer diagnosis is underemphasised as a chance to promote and encourage smoking cessation [13]. Cancer diagnosis may be viewed as an unsuitable time to discuss smoking due to the overburden of new treatment regimens and major life changes [13]. However, a recent study revealed that cancer patients are still willing and motivated to quit smoking after diagnosis [11, 14]. Nevertheless, based on previous population studies, most of the attempts to quit smoking seemed to be without assistance [15]. This emphasizes the need for smoking cessation support to strengthen cancer patients’ existing motivation.

According to the National Comprehensive Cancer Network (NCCN) Clinical Practice Guidelines, smoking cessation is recommended for cancer patients diagnosed at any stage [13, 15]. Based on a study conducted by Barnett *et al* [13] results showed that smoking cessation within 6 months of nonmetastatic solid cancer diagnosis led to longer survival and lower 3-year mortality when compared to no smoking cessation within 6 months. Nonetheless, the magnitude of the latter benefit is still unclear [13]. Moreover, other studies reported that smoking cessation is associated with a lower risk of all-cause mortality in patients diagnosed with lung cancer [15]. Evidence is also suggestive of a lower risk of head and neck cancers [7, 8]. Meanwhile, there is no clear proven association between quitting smoking and bladder cancer prognosis [7].

Although smoking cessation has proven benefits in improving quality of life and decreasing side effects of cancer treatment [8, 10], smoking cessation counselling is still not routinely provided in cancer care [7]. Physicians frequently face barriers in the attempt to successfully implement smoking cessation [16]. Some documented barriers include lack of knowledge or adequate training, lack of time and beliefs of the ineffectiveness of their interventions in addition to patients’ resistance to quitting smoking [11, 16]. The aim of this study is to examine how often physicians (particularly urologists, oncologists, radiation oncologists and family physicians) discuss smoking status and provide smoking cessation counselling or referral for cancer patients to smoking cessation programs; and to assess the barriers they face during such interventions.

## Methods

### Participants

This survey was conducted between June 2022 and January 2023. After receiving IRB approval from the American University of Beirut Medical Center, an e-mail containing a link to access the survey anonymously was sent to urologists, oncologists (including all subspecialties, surgical oncologists, ear nose and throat physicians), family medicine physicians and radiation oncologists who practice in Lebanon and deal with patients having smoking-related cancers (purposive sampling). In collaboration with the Lebanese Society of Medical Oncology (LSMO), Lebanese Urology Society (LUS) and Lebanese Society of Family Medicine (LSFM), e-mails reminding the target audience to fill out the survey were sent every 3 months for three consecutive times. Responses that were excluded from the analysis included incomplete surveys, surveys that were filled too rapidly or too slowly (required <1 minute or >30 minutes to be filled), and surveys with illogical or incomprehensive answers.

### Questionnaire

**Figure 1.**
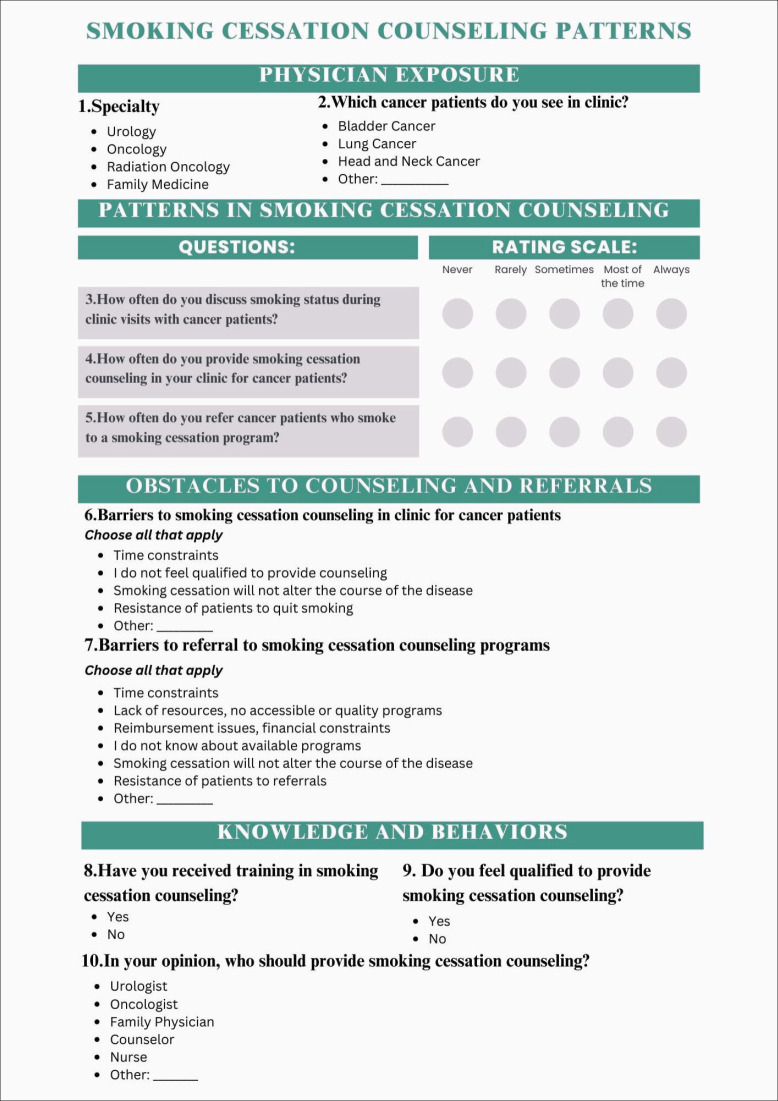
Questionnaire including the ten items with answer choices.

The electronic questionnaire is a self-administered, web-based (mobile-friendly), anonymous questionnaire that was developed on LimeSurvey and hosted safely and securely on the American University of Beirut (AUB) servers. The questionnaire consisted of ten questions related to smoking cessation counselling practices, barriers and attitudes, and requires an approximate time of 3–5 minutes to complete (Figure 1). The questions in sections 1, 2 and 4 (Physician exposure, patterns in smoking cessation counselling, knowledge and behaviours) were adapted from similar studies [17, 18]. In addition, to obtain the answer choices for the ‘obstacles to counselling and referrals’ section, a pilot sample of ten physicians that were later asked not to fill out the final survey was chosen (including medical oncologists, surgical oncologists, urologists, family medicine physicians, radiation oncologists and ear nose and throat surgeons). Two questions (What are barriers to smoking cessation counselling, and what are barriers to smoking cessation counselling referral among cancer patients) were asked of each physician and they were requested to respond open-endedly. Then, all responses were gathered, and the most frequently mentioned responses were included as an answer choice to questions 6 and 7. An ‘other’ response was added to allow participants to mention any obstacle that was not included in the answer choices.

### Data management and analysis

The collected data were stored on AUB servers. After completing data collection, the complete dataset was imported from LimeSurvey into IBM SPSS Statistics, v.26 (IBM Corp., Armonk, N.Y., USA). Statistical significance was set at the alpha level of 0.05. A descriptive analysis was performed on all ten items of the questionnaire, and associations between the different items were determined using the *χ*^2^ test.

## Results

847 physicians were e-mailed the questionnaire, 180 filled out the survey, and a total of 146 complete responses were obtained and used for analysis with a response rate of 17.23. Almost an equal number of participants among the four targeted specialties participated, with the highest response rate from family medicine physicians (29.5%). Out of the 39 oncologists who responded to the survey, 29 were medical oncologists who did not specify their subspecialty, 5 were surgical oncologists and 5 were ear, nose and throat surgeons. Almost all physicians ask about smoking status in cancer patients during their initial encounter, but only 45.9% provide smoking cessation counselling most of the time or always, and only 24% refer patients to smoking cessation counselling programs most of the time or always. Only 27.4% of all respondents have received formal smoking cessation training, and only those (27.4%) feel capable of providing smoking cessation counselling in the clinic. 54.1% of respondents believe that smoking cessation should be provided by a smoking cessation counselor (Table 1).

Among barriers to smoking cessation counselling in the clinic, the most reported barriers were the resistance of patients to quit smoking (45.9%), followed by time constraints (40.4%). And among barriers to referral to a smoking cessation counselling program, the most reported barriers were reimbursement issues (33.6%), and lack of knowledge about available smoking cessation programs (23.3%) (Table 1).

When comparing responses among the different specialties, we found that family medicine physicians were more likely to provide smoking cessation counselling in the clinic (69%), more likely to refer patients to a smoking cessation counselling program (44%), more likely to have received a formal smoking cessation counselling training (67%), and more likely to feel capable of providing smoking cessation counselling (93%), with *p*-values of 0.001, 0.001, 0.001 and 0.004, respectively.

Regarding barriers to smoking cessation counselling, Oncologists and family physicians were more likely to report patient-related factors (resistance of patients, depression and so on) as barriers (61% and 65%, respectively), whereas urologists and radiation oncologists were more likely to report physician-related factors (time constraints, not feeling competent and so on) as barriers (58% and 74%, respectively), *p*-value 0.005. A similar trend was seen regarding barriers to referral to smoking cessation counselling programs, whereby oncologists and family physicians were more likely to report patient-related factors (reimbursement issues, resistance of patients and so on.) as barriers (62% and 93%, respectively), while radiation oncologists were more likely to report physician-related factors (lack of knowledge about available programs and so on) as barriers (74%). Urologists reported both factors equally as much, *p*-value 0.001, (Table 2).

We also found that physicians who provide smoking cessation counselling in clinics are more likely to refer patients to a smoking cessation counselling program (*p*-value 0.021), and are more likely to have received smoking cessation counselling training (*p*-value 0.036).

## Discussion

Healthcare workers (HCWs), especially clinical providers, have a responsibility to educate their patients regarding the importance of smoking cessation. International organisations including the American Society of Clinical Oncology and the NCCN advocate the implementation of tobacco cessation services into clinical practice [19]. Our results highlight a striking deficiency in smoking cessation counselling and interventions in our region. Nevertheless, studies from different parts of the world found similar results; two studies investigating the use of the 5 A’s model by HCW (asking, advising, assessing, assisting or arranging follow-up) which has been proposed by evidence-based guidelines for smoking cessation [20] found that most HCWs perform the ‘Ask, Advice and Assess’, but rarely ‘Assist and Arrange follow-ups’ when approaching smokers [21, 22]. Weaver *et al* [18] reported that most oncologists report that they advise patients to quit smoking, however only 15%–30% provide interventions to assist their patients in smoking cessation. Along the same lines, our results reveal that there is a remarkable paucity of referrals to smoking cessation programs, which is most probably because HCWs consider this task to be tedious and time-consuming. Thus, having built-in automated referrals integrated with electronic health record (EHR)-based systems might serve as a helpful alternative to solve this problem in settings where EHRs are readily available [23, 24]. An alternative method in settings where EHRs are not available includes fax referrals which were shown to be less effective than electronic referrals, but nevertheless necessary [25]. In fact, it is important to mention that regardless of the referral method, patients who received referrals to a smoking cessation program were found in the literature to be highly likely to actually stop smoking on follow up [25, 26].

In our study, family physicians were more likely to provide smoking cessation counselling in the clinic and were also the most likely physicians to have received formal smoking cessation training. We also found that physicians who received formal training were more likely to educate patients about smoking cessation. This is probably because they learned, through their training, to integrate smoking cessation counselling as a routine procedure in their practice and thus became more comfortable and efficient at doing so. These findings are in concordance with the literature [27–30], whereby physicians who are trained are more likely to be comfortable providing counselling in the clinical setting. We speculate that training HCWs is important for smoking cessation; however, we still lack strong evidence that shows whether it leads to higher rates of quitting in the long term [31, 32].

The barriers that could possibly face physicians when tackling the issue of smoking habits during a patient encounter are numerous; however, in our study, patient-related factors represented the largest proportion of causes. In fact, many of the interviewed physicians believe that vulnerable patients, like cancer patients who are facing the burden of their cancer diagnosis, are not always good candidates for smoking cessation counselling. On the other hand, physicians may prioritise this topic in certain cases that permit it. For instance, in the literature, oncologists were found to shed more light on this issue when dealing with tobacco-related cancer cases, cases of recurrent cancer or cases where the prognosis is favorable [33].

In a large systematic review investigating the effect of smoking cessation counselling, it was found that patients who have received advice from their physician were 1.66 times more likely to quit smoking, and there was no significant difference between intensive and minimal advising methods of counselling [30]. This indicates that even a brief intervention is enough to encourage patients to stop smoking. Rose *et al* [34] studied the effect of smoking advice on an intervention group as compared to a control group after 20 years of follow up and found 7% lower mortality rates, 13% lower risk of fatal coronary heart disease and 11% lower risk of lung cancer in the intervention group, highlighting the reasonable yet indirect effect of smoking cessation counselling on disease-related outcomes. However, the results were not significant.

Furthermore, most of our participants argued that non-medical doctor (MD) HCWs should counsel the patients. This is probably because MD HCWs are already overwhelmed with a heavy workload. For this reason, it might be more efficient if this approach is changed from an individualistic to an interprofessional one, where more than one healthcare member, such as physicians, registered nurses and physician assistants are involved in the counselling and referral of patients when needed.

Our study represents the largest survey among physicians concerning smoking cessation counselling patterns in cancer patients in Lebanon. However, it is not without limitations. First, our questionnaire was sent by e-mail to physicians who treat smoking-related cancers and many subspecialties were under-represented (including ear, nose and throat physicians). Second, internal medicine physicians including pulmonologists were not included, which could affect our results in a meaningful way due to sampling bias. This indicates the need for future research including a larger and more diverse sample to enhance generalisability. Third, we acknowledge that asking about patterns in smoking cessation counselling could introduce recall and frequency bias. Finally, we acknowledge that our final questionnaire is not a validated questionnaire and was derived from a small pilot sample’s qualitative answers which could affect validity and generalisability.

## Conclusion

Our data reveals a deficiency in smoking cessation counselling and referral of cancer patients to smoking cessation counselling programs in our region. The effort needs to be put into educating physicians about available smoking cessation counselling programs and the development of more of these programs. Moreover, there is a need for studies investigating the direct correlation between smoking cessation counselling and disease-related outcomes in cancer patients.

## Conflicts of interest

The authors declare that they have no conflicts of interest regarding the material discussed in the manuscript.

## Funding

The authors report no involvement in the research by any sponsor that could have influenced the outcome of this work.

## Institutional review

The study was reviewed and approved by the Institutional Review Board of the American University of Beirut Faculty of Medicine before the survey was conducted.

## Author contributions

**JN:** Research Concept and Design, Collection and data assembly, Data analysis and Interpretation, Writing-Original Draft, Critical Revision of the article; **MH** Research Concept and Design, Collection and data assembly, Writing-Original Draft **AEA:** Research Concept and Design, Collection and data assembly, Writing-Original Draft; **NN:** Research Concept and Design, Collection and data assembly, Writing-Original Draft; **TT:** Research Concept and Design, Writing-Original Draft; **MR:** Research Concept and Design, Critical Revision of the Article; **AEH:** Research Concept and Design, Critical Revision of the Article; **DM:** Conceptualisation, Methodology, Writing-Review & Editing, Supervision. All authors read and approved the final version of the manuscript.

## Figures and Tables

**Table 1.** Distribution of answer choices to each of the survey’s ten questions.

Question	Answer choices	Frequency	Percent
Specialty	Oncology	39	26.7
	Family medicine	43	29.5
	Urology	36	24.7
	Radiation oncology	28	19.2
Smoking status discussion	Sometimes	4	2.7
	Most of the time/Always	142	97.3
Cessation counselling provided	Never/Rarely/Sometimes	79	54.1
	Most of the time/Always	67	45.9
Barriers to counselling	Time constraints	59	40.4
	I don't feel qualified to provide counselling	11	7.5
	Smoking cessation will not alter the course of the disease	1	0.7
	Resistance of patients to quit smoking	67	45.9
	All of the above	1	0.7
	terminal cancer	5	3.4
	Depression	2	1.4
Referral to cessation programs	Never/Rarely/Sometimes	111	76
	Most of the time/Always	35	24
Barriers to referral	Time constraints	4	2.7
	Lack of resources, no accessible and quality programs	13	8.9
	Reimbursement issues	49	33.6
	I don't know much about available smoking cessation programs	34	23.3
	Smoking cessation will not alter the course of the disease	1	0.7
	Patient's resistance to referrals	42	28.8
	All of the above	2	1.4
	Stress relief	1	0.7
Formal smoking cessation training	No	106	72.6
	Yes	40	27.4
Feels able to counsel	No	106	72.6
	Yes	40	27.4
Who should counsel	Oncologist	4	2.7
	Family physician	19	13
	Smoking cessation counselor	79	54.1
	Nurse	3	2.1
	Any HCW	27	18.5
	Any MD	13	8.9

MD: medical doctor

**Table 2.** Correlation between physician specialty and the distribution of answer choices for each of the questions.

Question	Answer choices	Specialty				*p*-value
		Onc *N* (%)	FM *N* (%)	Uro *N* (%)	Rad Onc *N* (%)	
Smoking status discussion	Never/Rarely/Sometimes	2 (5)	2 (5)	0 (0)	0 (0)	0.358
	Most of the time/Always	37 (95)	41 (95)	36 (100)	28 (100)	
Cessation counselling	Never/Rarely/Sometimes	23 (59)	13 (30)	22 (61)	21 (75)	0.001
	Most of the time/Always	16 (41)	30 (69)	14 (39)	7 (25)	
Barriers to counselling	Physician factors (time constraints, not competent, cessation will not change outcomes)	15 (40)	15 (35)	21 (58)	20 (74)	0.005
	Patient factors (resistance, depression, terminal cancer)	23 (61)	28 (65)	15 (42)	7 (26)	
Referral to program	Never/ Rarely/ Sometimes	28 (72)	24 (56)	32 (89)	27 (96)	0.001
	Most of the time/ Always	11 (28)	19 (44)	4 (11)	1 (4)	
Barriers to referral	Physician factors (time constraints, lack of programs, cessation will not change outcomes, don’t feel qualified)	15 (39)	3 (7)	18 (50)	16 (57)	0.001
	Patient factors (reimbursement, resistance, stress relief)	24 (62)	40 (93)	18 (50)	12 (43)	
Formal training	No	30 (77)	14 (33)	36 (100)	26 (93)	0.001
	Yes	9 (23)	29 (67)	0 (0)	2 (7)	
Able to counsel	No	13 (33)	3 (7)	15 (42)	8 (29)	0.004
	Yes	26 (67)	40 (93)	21 (58)	20 (71)	
Who should counsel	MD	8 (23)	13 (36)	4 (11)	6 (24)	0.001
	Non-MD HCW	26 (74)	10 (28)	28 (78)	18 (72)	
	Smoking cessation counselor	1 (3)	13 (36)	4 (11)	1 (4)	

Onc: Oncology; FM: Family medicine; Uro: Urology; Rad Onc: Radiation oncology; MD: medical doctor
